# Atypical Manifestations of Cat-Scratch Disease, United States, 2005–2014

**DOI:** 10.3201/eid2607.200034

**Published:** 2020-07

**Authors:** Courtney C. Nawrocki, Ryan J. Max, Natalie S. Marzec, Christina A. Nelson

**Affiliations:** Oak Ridge Institute for Science and Education, Oak Ridge, Tennessee, USA (C.C. Nawrocki, R.J. Max);; Centers for Disease Control and Prevention, Fort Collins, Colorado, USA (C.C. Nawrocki, R.J. Max, C.A. Nelson);; University of Colorado, Aurora, Colorado, USA (N.S. Marzec)

**Keywords:** cat-scratch disease, Bartonella henselae, bacteria, atypical manifestations, retinitis, meningitis/encephalitis, osteomyelitis, zoonoses, United States

## Abstract

Atypical manifestations that can be severe and difficult to diagnosis develop in 5%–20% of patients with cat-scratch disease. To clarify the epidemiology of atypical cat-scratch disease in the United States, we analyzed data from the 2005–2014 MarketScan national health insurance claims databases by using the International Classification of Diseases, 9th Revision, Clinical Modification, codes for cat-scratch disease and selected atypical manifestations: retinitis/neuroretinitis, conjunctivitis, neuritis, encephalitis, hepatosplenic disease, osteomyelitis, erythema nodosum, and endocarditis. Atypical cat-scratch disease accounted for 1.5% of all cases, resulting in an average annual incidence of 0.7 cases/100,000 population. Atypical cat-scratch disease was associated with increased risk for hospitalization (risk ratios 8.77, 95% CI 6.56–11.72) and occurred most often in female patients 10–14 years of age. Ocular (48.7%), hepatosplenic (24.6%), and neurologic (13.8%) manifestations were most common among patients. A more comprehensive understanding of atypical cat-scratch disease can improve patient diagnosis and potentially elucidate pathophysiology of the disease.

## Abstract 2

Atypical disease is most common among children and leads to increased risk for hospitalization.

Cat-scratch disease, a zoonotic bacterial infection, occurs worldwide and is caused by *Bartonella henselae*, a fastidious, intracellular gram-negative bacillus (*1*). Cats are the major reservoir of *B. henselae* and are infected by *Ctenocephalides felis* cat fleas. Although most cats infected with *B. henselae* are asymptomatic, signs such as fever and myocarditis might develop in some cats (*2*,*3*). Humans usually become infected through the scratches or bites of infected cats. *B. henselae* has also been shown to infect dogs (*4*), in some cases resulting in canine endocarditis (*5*–*7*). Although some human cases of cat-scratch disease have been linked to canine–human transmission (*8*–*12*), further research is needed to clarify the public health significance of *B. henselae* infection in dogs.

The true burden of cat-scratch disease in the United States is unknown because it is not a reportable condition; however, efforts have been made to estimate its incidence in the United States. In 1993, an analysis of hospital discharge data estimated a nationwide incidence of hospitalized cases of 0.77–0.86 cases/100,000 population annually (*13*). A subsequent study that examined a database of national health insurance claims during 2005–2013 found that incidence of cat-scratch disease in the United States was highest in southern states (6.4 cases/100,000 population) and in children 5–9 years of age (9.4 cases/100,000 population) (*14*).

Cat-scratch disease typically manifests as fever and an erythematous papule at the site of the cat scratch or bite, followed by lymphadenopathy in the regional lymph nodes that drain the area of inoculation (*15*). The papule usually appears 3–10 days after inoculation and can persist for several weeks, with regional lymphadenopathy developing 1–3 weeks postinoculation (*1*). From 80% to 95% of cases of cat-scratch disease are consistent with this typical presentation, and the remainder of cases manifest as atypical and more severe symptoms (*16*,*17*).

Atypical manifestations of cat-scratch disease can involve the eyes, nervous system, heart, liver, spleen, skin, or musculoskeletal system and might result in major illness (*1*,*15*). When cat-scratch disease involves the eye, the anterior compartment might be affected by Parinaud oculoglandular syndrome, and the posterior compartment might be affected by retinitis, retinochoroiditis, optic neuritis, uveitis, and vitritis (*18*–*20*). Nervous system involvement most commonly manifests as encephalopathy, but seizures, nerve palsies, neuritis, myelitis, and cerebellar ataxia have also been reported (*21*,*22*).

Endocarditis is more often seen in adults with cat-scratch disease than in children, although preexisting valvular disease puts children at increased risk for this complication (*1*). *Bartonella *infection can also cause abdominal pain and microabscesses in the liver and spleen (*23*), and in immunocompromised hosts can result in bacillary peliosis hepatis (*24*). In addition to the classic erythematous papule at the site of inoculation, erythema nodosum and bacillary angiomatosis are reported dermatologic manifestations of atypical infection (*25*,*26*). Osteolytic lesions, osteomyelitis, and arthritis have also been associated with cat-scratch disease (*16*,*24*,*26*). A study in 1998 found cat-scratch disease to be the third leading cause of prolonged fever of unknown origin in children, and a history of cat exposure was frequently absent (*27*).

Atypical manifestations of *B. henselae* infection can be severe, difficult to diagnose, and lead to lasting impairment. It is unclear why certain patients develop atypical cat-scratch disease, and little is known about its epidemiology. Improved understanding of atypical cat-scratch disease could lead to better recognition of cases by clinicians and inform efforts to understand the pathophysiology of this disease. The purpose of this study was to better characterize the rare and serious complications of this nonreportable zoonotic infection by using nationwide insurance claims data.

## Methods

To identify potential cases of atypical cat-scratch disease, we conducted a retrospective analysis of persons enrolled in the Truven Health MarketScan Commercial Claims and Encounters Database (Truven Health Analytics, https://www.ibm.com) during 2005–2014. The MarketScan Commercial Claims and Encounters Database includes persons <65 years of age covered by select employer-sponsored health insurance plans in all 50 states and contains administrative claims data on outpatient visits, inpatient admissions, and emergency department visits. Demographically, the MarketScan population generally mirrors the US population, with a slight overrepresentation of persons 50–59 years of age and a slight underrepresentation of persons 20–29 years of age (*28*).

Billing codes from outpatient, inpatient, and emergency department visits are assigned by either a clinician or billing specialist according to the International Classification of Diseases, 9th Revision, Clinical Modification (ICD-9-CM), and procedures are captured as either ICD-9-CM codes, Current Procedure Terminology codes, or Healthcare Common Procedure Coding System codes. Because the International Classification of Diseases, 10th Revision, Clinical Modification, was not officially adopted in the United States until 2015, those codes were not included.

We identified cat-scratch disease cases by extracting all enrollee visit records during the study period with an ICD-9-CM code for cat-scratch disease (078.3). The first instance of a 078.3 diagnosis code in a patient record was considered the index event. Atypical manifestations of interest were selected for analysis if they had recorded precedent in the literature as a complication of cat-scratch disease and distinct, clearly discernable ICD-9-CM codes associated with the specific manifestation. Based on these criteria, the known complications of cat-scratch disease included for analysis were endocarditis, osteomyelitis, erythema nodosum, conjunctivitis, retinitis/neuroretinitis, encephalitis, neuritis, and hepatosplenic disease. We included ICD-9-CM codes associated with optic neuritis in the retinitis/neuroretinitis category. Encounters with an ICD-9-CM code for cat-scratch disease and an accompanying diagnostic code to indicate the anatomic location of a wound or inoculation site for *B. henselae* were also flagged for analysis and were categorized as either head or neck region, arm or shoulder region, leg or hip region, or torso region. We compiled a detailed list of all ICD-9-CM codes used to identify atypical manifestations of cat-scratch disease (Appendix Table).

We extracted insurance billing records of enrollees with ICD-9-CM codes for cat-scratch disease and selected manifestations at either the same encounter or within a 30-day window of one another. These records were evaluated along with previous and subsequent records by 2 independent reviewers (R.J.M. and C.A.N.) to ensure that the clinical picture was consistent with the coded atypical manifestation based on diagnosis codes, procedure codes, and provider types. If plausible alternative causes of the selected manifestation or likely coding errors were identified, we did not include the enrollee record as an atypical case. In cases of discordance, a third reviewer (Paul Mead) determined final categorization based on record review.

We included persons with an ICD-9-CM code for cat-scratch disease but without accompanying atypical manifestation as typical cases for comparison. Residence in a rural area was assigned if an enrollee did not reside in a metropolitan statistical area, as designated by the US Office of Management and Budget. Because previous research has identified increases in cat-scratch disease in late summer, fall, and January (*13*,*14*,*29*), we categorized month of onset as either late summer and fall, January, or all other months for analysis.

We performed descriptive and comparative statistical analyses by using JMP version 13.2.1 (https://www.jmp.com) and SAS version 9.3 (https://www.sas.com). We used Pearson χ^2^ tests or Fisher exact tests for comparisons of categorical variables. To compare the conditional probability of having atypical cat-scratch disease across strata of potential variables of interest (e.g., sex, age category), we calculated risk ratios (RRs) and associated 95% CIs. Human subjects review at the Centers for Disease Control and Prevention determined that institutional review board approval was not required for this study.

## Results

### Study Population and Incidence

During 2005–2014, the MarketScan database contained a median of 44,488,485 (range 16,159,068–53,131,420) enrollees each year. Of 14,824 cat-scratch disease cases identified from MarketScan during this period, 224 (1.5%) cases were classified as atypical (Table 1). The average annual incidence of atypical cat-scratch disease diagnoses during the study period was 0.7 cases/100,000 population.

**Table 1:** Characteristics of patients with cat-scratch disease and risk factors for development of atypical cat-scratch disease, United States, 2005–2014

Characteristic	Typical disease, no. (%), n = 14,600	Atypical disease, no. (%), n = 224	Risk ratio (95% CI)*
Sex			
M	5,583 (38.2)	94 (42.0)	1.17 (0.90–1.52)
F	9,017 (61.8)	130 (58.0)	Referent
Age, y			
Child <14	4,678 (32.0)	81 (36.2)	1.20 (0.91–1.57)
Adult, 15–49	6,421 (44.0)	106 (47.3)	Referent
Adult, 50–64	3,501 (24.0)	37 (16.5)	0.63 (0.44–0.90)
Month of onset			
Late summer and fall†	5,470 (37.5)	93 (41.5)	1.18 (0.90–1.56)
January	1,490 (10.2)	22 (9.8)	1.03 (0.65–1.64)
All other months‡	7,640 (52.3)	109 (48.7)	Referent
Hospitalized	487 (3.3)	56 (25)	8.77 (6.56–11.72)
Residence in southern state	7,732 (53.0)	129 (57.6)	1.20 (0.93–1.57)
Residence in rural area	3,235 (22.1)	51 (22.8)	1.06 (0.78–1.45)

*Outcome for risk ratio calculations was development of atypical cat-scratch disease. †August, September, October, and November. ‡February, March, April, May, June, July, and December.

Atypical cat-scratch disease was most common among adults 15–49 years of age (47.3%), and patients with atypical cat-scratch disease were more likely to be hospitalized than those with typical manifestations (p<0.0001). 

### Distribution by Age and Sex

**Figure 1:**
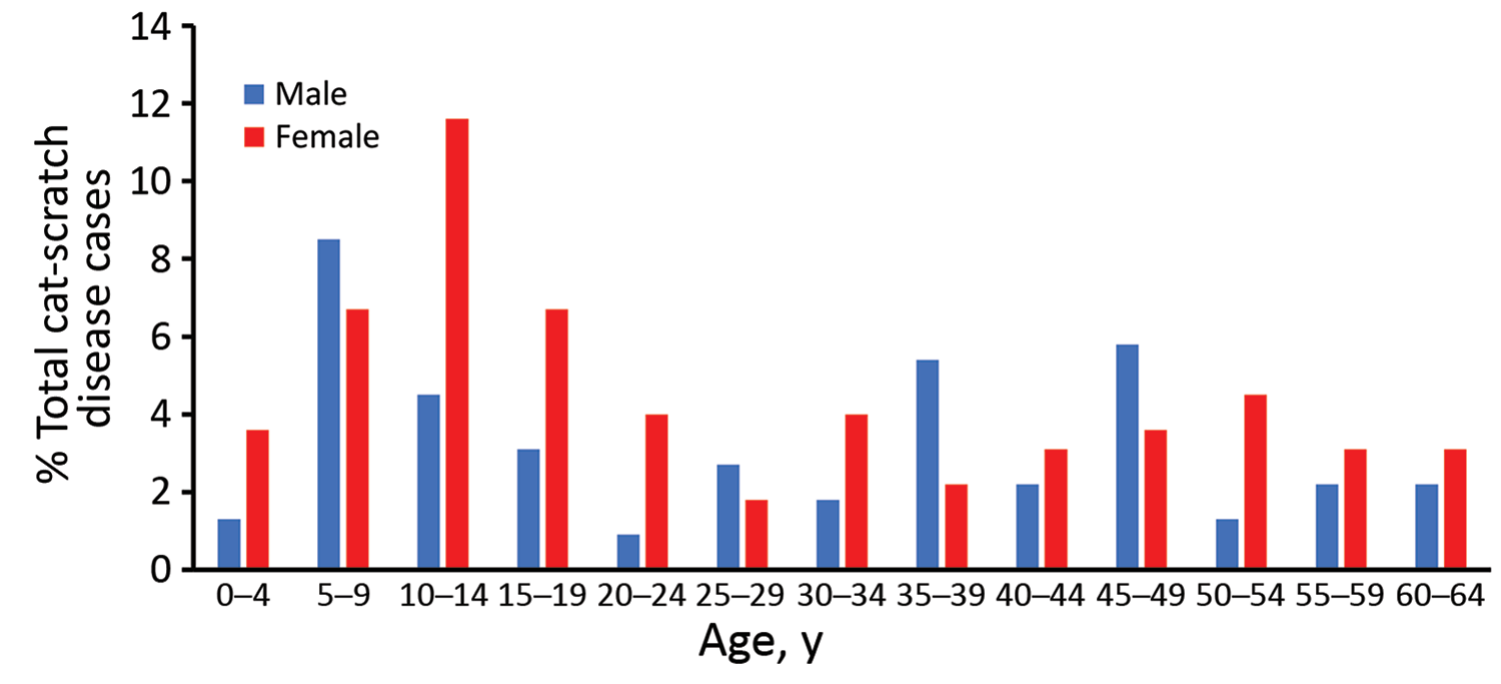
Age and sex distribution of patients with atypical cat-scratch disease, United States, 2005–2014.

Children <14 years of age accounted for 36.2% of atypical cat-scratch disease diagnoses overall; 26 cases (11.4%) were in female patients 10–14 years of age (Figure 1). Among female patients 10–14 years of age, 16 patients (61.5%) had ocular manifestations (13 retinitis/neuroretinitis and 3 conjunctivitis), and 6 patients (23.1%) had hepatosplenic disease.

Nearly half of all patients with atypical cat-scratch disease were younger adults (15–49 years of age). When we compared older adults with younger adults, older adults (50–64 years of age) had a decreased risk for having atypical cat-scratch disease (RR 0.63, 95% CI 0.44–0.90) (Table 1).

### Seasonality

**Figure 2:**
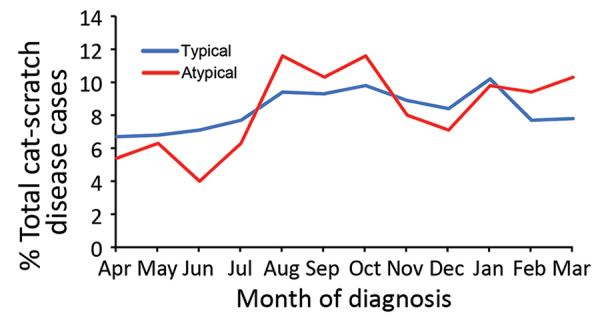
Seasonal variation of atypical and typical cat-scratch disease by month of diagnosis, United States, 2005–2014.

Atypical cat-scratch disease diagnoses increased from August through March, and diagnoses were concentrated during August–October (33.5% of diagnoses) and January–March (29.5% of diagnoses) (Figure 2), although neither diagnosis in late summer and fall or diagnosis in January were found to be risk factors for development of atypical cat-scratch disease (Table 1). Trends in atypical cat-scratch disease diagnoses were similar to trends in typical cat-scratch disease diagnoses. However, typical cat-scratch disease had less defined peak periods, and diagnoses decreased sharply after January.

### Geographic Distribution and Residence in Rural Area

**Figure 3:**
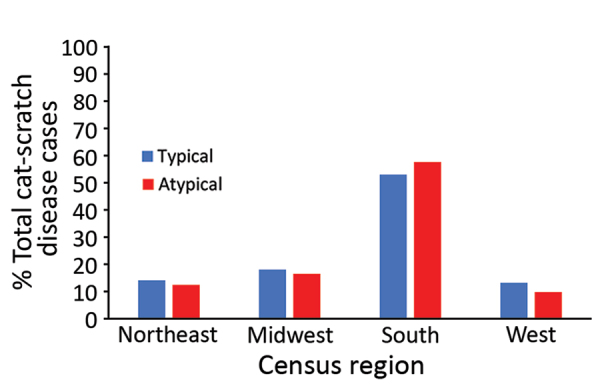
Proportions of typical and atypical cat-scratch disease by US Census region, United States, 2005–2014. Northeast: Connecticut, Maine, Massachusetts, New Hampshire, Rhode Island, Vermont, New Jersey, New York, Pennsylvania. Midwest: Illinois, Indiana, Iowa, Kansas, Michigan, Minnesota, Missouri, Nebraska, North Dakota, South Dakota, Ohio, Wisconsin. South: Arkansas, Delaware, Florida, Georgia, Louisiana, Maryland, North Carolina, Oklahoma, South Carolina, Texas, Virginia, West Virginia, Alabama, Hawaii, Kentucky, Mississippi, Oregon, Tennessee. West: Alaska, Arizona, California, Colorado, Idaho, Montana, Nevada, New Mexico, Utah, Washington, Wyoming, Puerto Rico, Virgin Islands.

Most (57.6%) cases of atypical cat-scratch disease occurred in the southern region of the United States (57.6%), followed by the midwest (16.5%) and northeast (12.5%) regions (Figure 3). The geographic distribution of atypical cases did not differ significantly from cases of typical cat-scratch disease.

Residence in a rural area was not a risk factor for development of atypical cat-scratch disease (RR 1.06, 95% CI 0.78–1.45). Also, the proportion of patients with atypical cat-scratch disease living in a rural area did not differ from the proportion of patients with typical cat-scratch disease living in a rural area (p = 0.70).

### Atypical Manifestations by Type

Among 224 patients with atypical cat-scratch disease, 109 (48.7%) had ocular manifestations (retinitis/neuroretinitis or conjunctivitis), 55 (24.6%) had hepatosplenic disease, and 31 (13.8%) had neurologic manifestations (neuritis or encephalitis). The remaining 33 (14.7%) case-patients had osteomyelitis, erythema nodosum, or endocarditis (Table 2). Among patients with ocular manifestations, 82.6% had retinitis/neuroretinitis; most (64.5%) patients with neurologic manifestations had encephalitis. Three (1.3%) patients with atypical cat-scratch disease had >1 manifestation: 1 patient with osteomyelitis and hepatosplenic disease; 1 patient with endocarditis and hepatosplenic disease; and 1 patient with osteomyelitis, encephalitis, and hepatosplenic disease.

**Table 2:** Demographic characteristics for patients by manifestation of atypical cat-scratch disease, United States, 2005–2014

Characteristic	No. (%)	Sex, no. (%)			Age category, y, no. (%)			Hospitalized, no. (%)
		M	F		0–14	15–49	50–64	
Atypical disease	224*	94 (42.0)	130 (58.0)		81 (36.2)	106 (47.3)	37 (16.5)	56 (25.0)
Ocular disease	109 (48.7)	46 (48.9)	63 (48.5)		33 (40.7)	60 (56.6)	16 (43.2)	10 (17.9)
Retinitis/neuroretinitis	90 (40.2)	36 (38.3)	54 (41.5)		23 (28.4)	53 (50.0)	14 (37.8)	8 (14.3)
Conjunctivitis	19 (8.5)	10 (10.6)	9 (6.9)		10 (12.3)	7 (6.6)	2(5.4)	2 (3.6)
Hepatosplenic disease	55 (24.6)	24 (25.5)	31 (23.8)		25 (30.9)	21 (19.8)	9 (24.3)	24 (42.9)
Neurologic disease	31 (13.8)	13 (13.8)	18 (13.8)		12 (14.8)	13 (12.3)	6 (16.2)	13 (23.2)
Encephalitis	20 (8.9)	12 (12.8)	8 (6.2)		12 (14.8)	8 (7.5)	0 (0)	13 (23.2)
Neuritis	11 (4.9)	1 (1.1)	10 (7.7)		0 (0)	5 (4.7)	6 (16.2)	0 (0)
Osteomyelitis	14 (6.3)	6 (6.4)	8 (6.2)		9 (11.1)	4 (3.8)	1 (2.7)	7 (12.5)
Erythema nodosum	11 (4.9)	2 (2.1)	9 (6.9)		4 (4.9)	5 (4.7)	2 (5.4)	4 (7.1)
Endocarditis	8 (3.6)	4 (4.3)	4 (3.1)		1 (1.2)	4 (3.8)	3 (8.1)	2 (3.6)

*A total of 228 manifestations of atypical cat-scratch disease were seen among 224 patients. Three patients had >1 manifestation; 1 patient had osteomyelitis and hepatosplenic disease;1 patient had endocarditis and hepatosplenic disease; and 1 patient had osteomyelitis, encephalitis, and hepatosplenic disease.

Children <14 years of age were at increased risk for hepatosplenic disease (RR 1.76, 95% CI 1.04–2.99) and osteomyelitis (RR 3.81, 95% CI 1.28–11.37) compared with persons >15 years of age. Older adults (50–64 years of age) were less likely to show development of ocular manifestations (retinitis/neuroretinitis and conjunctivitis) than younger adults (15–49 years of age) (RR 0.49, 95% CI 0.28–0.85).

**Figure 4:**
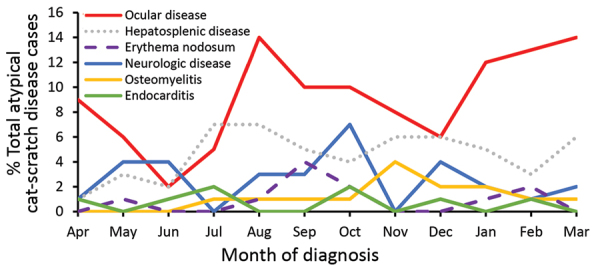
Seasonal variation of atypical cat-scratch disease manifestations by month of diagnosis, United States, 2005–2014.

Among persons with ocular (retinitis/neuroretinitis and conjunctivitis) manifestations, most diagnoses were made during August–October (31.2%) and January–March (35.8%). Among persons with neurologic (neuritis and encephalitis) manifestations, diagnoses were concentrated during October (22.6%). We observed no notable trends in seasonality of diagnoses for other manifestations of atypical cat-scratch disease (Figure 4). We also observed no differences in geographic distribution or rurality by manifestation of atypical cat-scratch disease.

### Hospitalization of Atypical Case-Patients

Patients with atypical cat-scratch disease were more likely to be hospitalized than patients with typical cat-scratch disease (RR 8.77, 95% CI 6.56–11.72) (Table 1). Among patients with atypical cat-scratch disease, children <14 years of age accounted for 60.7% of hospitalizations and had an increased risk for hospitalization compared with adults 15–49 years of age (RR 2.34, 95% CI 1.44–3.79). A total of 57.1% of the hospitalizations occurred during August–November, and we found an overall increased risk for hospitalization during this period when compared with all other months, except for January (RR 1.88, 95% CI 1.15–3.05) (Table 3).

**Table 3:** Demographic characteristics for patients hospitalized with atypical cat-scratch disease and associated risk factors for hospitalization, United States, 2005–2014

Characteristic	Hospitalized, no. (%), n = 56	Not hospitalized, no. (%), n = 168	Risk ratio (95% CI)
Sex			
M	26 (46.4)	68 (40.5)	1.19 (0.76–1.89)
F	30 (53.6)	100 (59.5)	Referent
Age, y			
Child <14	34 (60.7)	47 (28.0)	2.34 (1.44–3.79)
Younger adult, 15–49	19 (33.9)	87 (51.8)	Referent
Older adult, 50–64	3 (5.4)	34 (20.2)	0.45 (0.14–1.44)
Month of onset			
Late summer and fall*	32 (57.1)	61 (36.3)	1.88 (1.15–3.05)
January	4 (7.1)	18 (10.7)	0.99 (0.38–2.62)
All other months†	20 (35.7)	89 (53.0)	Referent
Residence in southern state	36 (64.3)	93 (55.4)	1.33 (0.82–2.14)
Residence in rural area	11 (19.6)	40 (23.8)	1.26 (0.71–2.26)

*August, September, October, and November. †February, March, April, May, June, July, and December.

Increased risks of hospitalization were found for neurologic manifestations (neuritis and encephalitis) (RR 1.88, 95% CI 1.15–3.08), hepatosplenic disease (RR 2.30, 95% CI 1.49–3.55), and osteomyelitis (RR 2.13, 95% CI 1.20–3.82). Ocular manifestations (retinitis/neuroretinitis and conjunctivitis) were associated with decreased risk for hospitalization (RR 0.23, 95% CI 0.12–0.43).

### Location of Wound

Information on wound location was available for only 10 (4.5%) patients with atypical cat-scratch disease. Among these persons, 2 with conjunctivitis, 1 with encephalitis, and 2 with hepatosplenic disease had a wound on the head or neck; 1 with osteomyelitis and 3 with hepatosplenic manifestations had a wound on the arm or shoulder; 1 with endocarditis had a wound on the leg or hip; and 1 with endocarditis had a wound on an unspecified limb.

## Discussion

Using US nationwide insurance claims data, we identified and characterized 224 atypical cases of cat-scratch disease during 2005–2014 and estimated an average annual incidence of 0.7 cases/100,000 population. Nearly half of all atypical cat-scratch disease cases had ocular manifestations, most of which were retinitis/neuroretinitis. Atypical cat-scratch disease was most prevalent among female patients 10–14 years of age, who most commonly had ocular manifestations.

Trends in hospitalizations of patients with cat-scratch disease highlight the severity of atypical cat-scratch disease compared with typical cat-scratch disease. Atypical cat-scratch disease appears to be particularly severe among children <14 years of age, who had an increased risk for hospitalization. Adults 50–64 years of age had the lowest risk for development of atypical cat-scratch disease and specifically ocular manifestations. Reasons that older adults might have complications associated with cat-scratch disease less often than other age groups are unclear and require further study.

Severity of cat-scratch disease in children has been previously documented. In a study conducted by Reynolds et al., ≈25% of hospitalizations of children for cat-scratch disease were caused by complications associated with atypical cat-scratch disease; neurologic and hepatosplenic complications were most common (*30*). Although children in our study were also particularly at risk for hepatosplenic disease, neurologic and hepatosplenic complications were associated with increased risk for hospitalization in our overall study population, indicating that these manifestations are particularly severe for all age groups. Encephalitis was the most common neurologic manifestation in our population, which was also consistent with findings of Reynolds et al., in which most hospitalizations of children for neurologic complications of cat-scratch disease were caused by encephalitis or encephalopathy (*30*). Thus, physicians should consider cat-scratch disease in patients who have encephalitis or new onset hepatosplenic abnormalities, especially children.

Previous studies have documented the highest rates of cat-scratch disease in late summer and fall and a separate peak often seen in January (*13*,*14*,*29*). One such study found that rates of *B. henselae* seropositivity among samples submitted to Mayo Clinic Laboratories over a 10-year period were highest during September–January, with the highest annual rates in January (*29*). Typical cat-scratch disease diagnoses in our study followed similar seasonal patterns to those previously reported. However, atypical cat-scratch disease appeared more concentrated during August–October and January–March. The reasons for this finding are unclear but might include delays in diagnosis of atypical cat-scratch disease. For example, patients who contract cat-scratch disease and had complications during January might not be given a diagnosis of atypical cat-scratch disease at that time because they do not show classic symptoms or their symptoms take time to develop and care-seeking is delayed.

Furthermore, a recent case series of ocular manifestations of cat-scratch disease reported that 9 of 10 patients had symptoms <3 months before showing development of ocular complications and that 3 patients had been originally given misdiagnoses of etiologies other than cat-scratch disease (*31*). Given that ocular manifestations of cat-scratch disease were most common in our study, increased diagnoses of atypical cat-scratch disease through March could be a sign of delayed diagnoses, particularly for manifestations that are less severe, such as those involving the eye.

Trends related to geographic distribution of cases did not differ between atypical and typical cat-scratch disease. Similar to findings from 3 previous studies that reported the highest incidences of cat-scratch disease in the southern United States (*13*,*14*,*30*), in our study, most typical (53.0%) and atypical (57.6%) cat-scratch disease cases occurred in patients residing in this region. In addition, a national survey of US healthcare providers found that those in the Pacific and southern regions of the United States were more likely to have been given a diagnosis of cat-scratch disease than in other regions (*32*). These findings are further supported by studies that have found higher average *B. henselae* seroprevalences and active bacteremia in pet cats from warmer, more humid climates, including the southern United States (*33*,*34*). Thus, healthcare providers in regions with climates that support flea abundance should be aware of the risk for cat-scratch disease and be able to recognize its atypical manifestations.

This study had several limitations. First, although MarketScan is a large database of insurance claims data from persons covered by employer-sponsored insurance, it is a convenience sample and may not accurately represent the characteristics of all persons in the United States. For example, trends we see in atypical cat-scratch disease by geographic region and rural residence might be biased by differences in coverage and access to care that are not accounted for here. Furthermore, MarketScan does not include data for adults >65 years of age, military personnel, uninsured persons, or Medicaid/Medicare enrollees. These specific populations might show varying degrees of cat-scratch disease severity or risk that are not captured in our results. In addition, because only persons <65 years of age are included in the database, the proportion of children who have cat-scratch disease might be artificially inflated. The number of patients who had atypical cat-scratch disease was small, especially when broken down by manifestation. Thus, it is difficult to draw conclusions regarding risk factors for specific manifestations of atypical cat-scratch disease and hospitalization within these groups.

Furthermore, misclassification could have occurred when ICD-9-CM codes were used to classify atypical cat-scratch disease for several reasons. ICD-9-CM codes are subject to error from the clinicians and billing specialists who enter them. In addition, we excluded records that fit our criteria for manifestations of atypical cat-scratch disease but lacked additional supporting information, which could have caused us to underestimate the true burden of atypical cat-scratch disease. Last, codes for some known atypical cat-scratch disease manifestations, such as pulmonary complications and thrombocytopenia, were excluded because of etiologic ambiguity in enrollee records.

In conclusion, our findings indicate that atypical cat-scratch disease in the United States follows trends similar to those for typical cat-scratch disease but is more prevalent and severe among children <14 years of age and is least likely to occur in older adults (50–64 years of age). In addition, differences in seasonality of diagnoses were seen, which might be an indication that diagnosis of atypical cat-scratch disease is often delayed. Ocular (retinitis/neuroretinitis and conjunctivitis) and hepatosplenic complications were the most common manifestations of atypical cat-scratch disease. Improved understanding of atypical cat-scratch disease might lead to better recognition of cases by clinicians, as well as inform efforts to clarify the pathophysiology of this disease.

**Appendix.** Additional information on atypical manifestations of cat-scratch disease, United States, 2005–2014.
