# Supplementary material for: Atypical Manifestations of Cat-Scratch Disease, United States, 2005–2014
Source: Emerg Infect Dis. 2020 Jul;26(7):1438–46. doi: 10.3201/eid2607.200034 (PMC7323523; doi:10.3201/eid2607.200034)
Supplement: Supplementary file 1 — Appendix. Additional information on atypical manifestations of cat-scratch disease, United States, 2005–2014. [file 20-0034-Techapp-s1.pdf]

Article DOI: https://doi.org/10.3201/eid2607.200034

Atypical Manifestations of Cat-Scratch Disease, United States, 2005–2014

Appendix

Appendix Table. ICD-9-CM codes and descriptions used to identify atypical manifestations of cat-scratch disease*

ICD-9-CM code	Description
3689	Unspecified visual disturbance
22803	Hemangioma of retina
36011	Sympathetic uveitis
36100	Retinal detachment with retinal defect, unspecified
36101	Recent retinal detachment, partial, with single defect
36102	Recent retinal detachment, partial, with multiple defects
36103	Recent retinal detachment, partial, with giant tear
36104	Recent retinal detachment, partial, with retinal dialysis
36105	Recent retinal detachment, total or subtotal
3612	Serous retinal detachment
36189	Other forms of retinal detachment
3619	Unspecified retinal detachment
36213	Changes in vascular appearance of retina
36229	Other nondiabetic proliferative retinopathy
36230	Retinal vascular occlusion, unspecified
36254	Macular cyst, hole, or pseudohole
36281	Retinal hemorrhage
36282	Retinal exudates and deposits
36283	Retinal edema
36289	Other retinal disorders
3629	Unspecified retinal disorder
36321	Pars planitis
36370	Choroidal detachment, unspecified
36371	Serous choroidal detachment
36372	Hemorrhagic choroidal detachment
3638	Other disorders of choroid
3639	Unspecified disorder of choroid
36405	Hypopyon
36901	Better eye: total vision impairment; lesser eye: total vision impairment
36902	Better eye: near-total vision impairment; lesser eye: not further specified
36903	Better eye: near-total vision impairment; lesser eye: total vision impairment
36904	Better eye: near-total vision impairment; lesser eye: near-total vision impairment
36905	Better eye: profound vision impairment; lesser eye: not further specified
36906	Better eye: profound vision impairment; lesser eye: total vision impairment
36907	Better eye: profound vision impairment; lesser eye: near-total vision impairment
36908	Better eye: profound vision impairment; lesser eye: profound vision impairment
36910	Moderate or severe impairment, better eye, impairment level not further specified
36911	Better eye: severe vision impairment; lesser eye: blind, not further specified
36912	Better eye: severe vision impairment; lesser eye: total vision impairment
36913	Better eye: severe vision impairment; lesser eye: near-total vision impairment
36914	Better eye: severe vision impairment; lesser eye: profound vision impairment
36915	Better eye: moderate vision impairment; lesser eye: blind, not further specified
36916	Better eye: moderate vision impairment; lesser eye: total vision impairment
36917	Better eye: moderate vision impairment; lesser eye: near-total vision impairment
36918	Better eye: moderate vision impairment; lesser eye: profound vision impairment
36920	Moderate or severe impairment, both eyes, impairment level not further specified
36921	Better eye: severe vision impairment; lesser eye; impairment not further specified
36922	Better eye: severe vision impairment; lesser eye: severe vision impairment
36923	Better eye: moderate vision impairment; lesser eye: impairment not further specified
36924	Better eye: moderate vision impairment; lesser eye: severe vision impairment

ICD-9-CM code	Description
36925	Better eye: moderate vision impairment; lesser eye: moderate vision impairment
3693	Unqualified visual loss, both eyes
36960	Profound impairment, one eye, impairment level not further specified
36961	One eye: total vision impairment; other eye: not specified
36962	One eye: total vision impairment; other eye: near-normal vision
36963	One eye: total vision impairment; other eye: normal vision
36964	One eye: near-total vision impairment; other eye: vision not specified
36965	One eye: near-total vision impairment; other eye: near-normal vision
36966	One eye: near-total vision impairment; other eye: normal vision
36967	One eye: profound vision impairment; other eye: vision not specified
36968	One eye: profound vision impairment; other eye: near-normal vision
36969	One eye: profound vision impairment; other eye: normal vision
36970	Moderate or severe impairment, one eye, impairment level not further specified
36971	One eye: severe vision impairment; other eye: vision not specified
36972	One eye: severe vision impairment; other eye: near-normal vision
36973	One eye: severe vision impairment; other eye: normal vision
36974	One eye: moderate vision impairment; other eye: vision not specified
36975	One eye: moderate vision impairment; other eye: near-normal vision
36976	One eye: moderate vision impairment; other eye: normal vision
3698	Unqualified visual loss, one eye
3699	Unspecified visual loss
37221	Angular blepharoconjunctivitis
37222	Contact blepharoconjunctivitis
37289	Other disorders of conjunctiva
3729	Unspecified disorder of conjunctiva
37700	Papilledema, unspecified
37702	Papilledema associated with decreased ocular pressure
37703	Papilledema associated with retinal disorder
37732	Retrobulbar neuritis (acute)
37742	Hemorrhage in optic nerve sheaths
37749	Other disorders of optic nerve
3688	Other specified visual disturbances
4387	Late effects of cerebrovascular disease, disturbances of vision
37239	Other conjunctivitis
3410	Neuromyelitis optica
3630	Focal chorioretinitis and focal retinochoroiditis (fill)
3631	Disseminated chorioretinitis and disseminated retinochoroiditis (fill)
3632	Other and unspecified forms of chorioretinitis and retinochoroiditis (fill)
3684	Visual field defects (fill)
3725	Conjunctival degenerations and deposits (fill)
3770	Papilledema (fill)
3771	Optic atrophy (fill)
3772	Other disorders of optic disc (fill)
3773	Optic neuritis (fill)
3790	Scleritis and episcleritis (fill)
3792	Disorder of vitreous body (fill)
3799	Unspecified disorder of eye and adnexa (fill)
36900	Profound impairment, both eyes, impairment level not further specified
36012	Panuveitis
36212	Exudative retinopathy
36231	Central retinal artery occlusion
36232	Retinal arterial branch occlusion
36233	Partial retinal arterial occlusion
36234	Transient retinal arterial occlusion
36235	Central retinal vein occlusion
36236	Venous tributary (branch) occlusion
36241	Central serous retinopathy
36300	Focal chorioretinitis, unspecified
36301	Focal choroiditis and chorioretinitis, juxtapapillary
36303	Focal choroiditis and chorioretinitis of other posterior pole
36304	Focal choroiditis and chorioretinitis, peripheral
36305	Focal retinitis and retinochoroiditis, juxtapapillary
36306	Focal retinitis and retinochoroiditis, macular or paramacular
36307	Focal retinitis and retinochoroiditis of other posterior pole
36308	Focal retinitis and retinochoroiditis, peripheral
36310	Disseminated chorioretinitis, unspecified
36311	Disseminated choroiditis and chorioretinitis, posterior pole
36312	Disseminated choroiditis and chorioretinitis, peripheral
36313	Disseminated choroiditis and chorioretinitis, generalized

ICD-9-CM code	Description
36314	Disseminated retinitis and retinochoroiditis, metastatic
36315	Disseminated retinitis and retinochoroiditis, pigment epitheliopathy
36403	Secondary iridocyclitis, infectious
37600	Acute inflammation of orbit, unspecified
37701	Papilledema associated with increased intracranial pressure
372	Disorders of conjunctiva
3720	Acute conjunctivitis (fill)
37201	Serous conjunctivitis, except viral
37202	Acute follicular conjunctivitis
37203	Other mucopurulent conjunctivitis
37204	Pseudomembranous conjunctivitis
3722	Blepharoconjunctivitis, unspecified
37220	Blepharoconjunctivitis, unspecified
3723	Other and unspecified conjunctivitis (fill)
37230	Conjunctivitis, unspecified
37261	Granuloma of conjunctiva
3773	Optic neuritis (fill)
37730	Optic neuritis, unspecified
37739	Other optic neuritis
36320	Chorioretinitis, unspecified
7301	Chronic osteomyelitis (fill)
73010	Chronic osteomyelitis, site unspecified
73011	Chronic osteomyelitis, shoulder region
73012	Chronic osteomyelitis, upper arm
73013	Chronic osteomyelitis, forearm
73014	Chronic osteomyelitis, hand
73015	Chronic osteomyelitis, pelvic region and thigh
73016	Chronic osteomyelitis, lower leg
73017	Chronic osteomyelitis, ankle and foot
73018	Chronic osteomyelitis, other specified sites
73019	Chronic osteomyelitis, multiple sites
73030	Periostitis, without mention of osteomyelitis, site unspecified
73031	Periostitis, without mention of osteomyelitis, shoulder region
73032	Periostitis, without mention of osteomyelitis, upper arm
73033	Periostitis, without mention of osteomyelitis, forearm
73034	Periostitis, without mention of osteomyelitis, hand
73035	Periostitis, without mention of osteomyelitis, pelvic region and thigh
73036	Periostitis, without mention of osteomyelitis, lower leg
73037	Periostitis, without mention of osteomyelitis, ankle and foot
73038	Periostitis, without mention of osteomyelitis, other specified sites
73039	Periostitis, without mention of osteomyelitis, multiple sites
73000	Acute osteomyelitis, site unspecified
73001	Acute osteomyelitis, shoulder region
73002	Acute osteomyelitis, upper arm
73003	Acute osteomyelitis, forearm
73004	Acute osteomyelitis, hand
73007	Acute osteomyelitis, ankle and foot
73009	Acute osteomyelitis, multiple sites
73022	Unspecified osteomyelitis, upper arm
73023	Unspecified osteomyelitis, forearm
73024	Unspecified osteomyelitis, hand
73027	Unspecified osteomyelitis, ankle and foot
73029	Unspecified osteomyelitis, multiple sites
37603	Orbital osteomyelitis
73005	Acute osteomyelitis, pelvic region and thigh
73028	Unspecified osteomyelitis, other specified sites
73025	Unspecified osteomyelitis, pelvic region and thigh
73008	Acute osteomyelitis, other specified sites
73021	Unspecified osteomyelitis, shoulder region
73026	Unspecified osteomyelitis, lower leg
7302	Unspecified osteomyelitis
73020	Unspecified osteomyelitis, site unspecified
73005	Acute osteomyelitis, pelvic region and thigh
73028	Unspecified osteomyelitis, other specified sites
73025	Unspecified osteomyelitis, pelvic region and thigh
73006	Acute osteomyelitis, lower leg
73008	Acute osteomyelitis, other specified sites
73021	Unspecified osteomyelitis, shoulder region
73026	Unspecified osteomyelitis, lower leg

ICD-9-CM code	Description
73020	Unspecified osteomyelitis, site unspecified
4378	Other ill-defined cerebrovascular disease
4379	Unspecified cerebrovascular disease
2930	Delirium due to conditions classified elsewhere
2931	Subacute delirium
29389	Other specified transient mental disorders due to conditions classified elsewhere, other
2939	Unspecified transient mental disorder in conditions classified elsewhere
2948	Other persistent mental disorders due to conditions classified elsewhere
2949	Unspecified persistent mental disorders due to conditions classified elsewhere
3129	Unspecified disturbance of conduct
3207	Meningitis in other bacterial diseases classified elsewhere
32082	Meningitis due to gram-negative bacteria, not elsewhere classified
32089	Meningitis due to other specified bacteria
3209	Meningitis due to unspecified bacterium
3220	Nonpyogenic meningitis
32342	Other myelitis due to other infections classified elsewhere
32361	Infectious acute disseminated encephalomyelitis
32362	Other postinfectious encephalitis and encephalomyelitis
32363	Postinfectious myelitis
32382	Other causes of myelitis
3239	Unspecified causes of encephalitis, myelitis, and encephalomyelitis
32710	Organic hypersomnia, unspecified
32711	Idiopathic hypersomnia with long sleep time
32712	Idiopathic hypersomnia without long sleep time
32713	Recurrent hypersomnia
32714	Hypersomnia due to medical condition classified elsewhere
32719	Other organic hypersomnia
3332	Myoclonus
3449	Paralysis, unspecified
3568	Other specified idiopathic peripheral neuropathy
3570	Acute infective polyneuritis
78093	Memory loss
7811	Disturbances of sensation of smell and taste
7812	Abnormality of gait
7813	Lack of coordination
7814	Transient paralysis of limb
7961	Abnormal reflex
78039	Other convulsions
342	Flaccid hemiplegia
4374	Cerebral arteritis
345	Infantile spasms
436	Acute, but ill-defined, cerebrovascular disease
32341	Other encephalitis and encephalomyelitis due to other infections classified elsewhere
32381	Other causes of encephalitis and encephalomyelitis
37701	Papilledema associated with increased intracranial pressure
3483	Encephalopathy, not elsewhere classified (fill)
34830	Encephalopathy, unspecified
34839	Other encephalopathy
78097	Altered mental status
78002	Transient alteration of awareness
78009	Other alteration of consciousness
7800	Alteration of consciousness (fill)
780	General symptoms
7244	Thoracic or lumbosacral neuritis or radiculitis, unspecified
7292	Neuralgia, neuritis, and radiculitis, unspecified
7234	Brachial neuritis or radiculitis NOS
3343	Other cerebellar ataxia
3344	Cerebellar ataxia in diseases classified elsewhere
3412	Acute (transverse) myelitis
78931	Abdominal or pelvic swelling, mass, or lump, right upper quadrant
78941	Abdominal rigidity, right upper quadrant
78961	Abdominal tenderness, right upper quadrant
2895	Other diseases of spleen (fill)
28950	Disease of spleen, unspecified
28951	Chronic congestive splenomegaly
28953	Neutropenic splenomegaly
28959	Other diseases of spleen
570	Acute and subacute necrosis of liver
5719	Unspecified chronic liver disease without mention of alcohol

ICD-9-CM code	Description
5732	Hepatitis in other infectious diseases classified elsewhere
5733	Hepatitis, unspecified
5734	Hepatic infarction
5735	Hepatopulmonary syndrome
5738	Other specified disorders of liver
5739	Unspecified disorder of liver
7590	Anomalies of spleen
7904	Nonspecific elevation of levels of transaminase or lactic acid dehydrogenase
7905	Other nonspecific abnormal serum enzyme levels
7948	Nonspecific abnormal results of function study of liver
78901	Abdominal pain, right upper quadrant
28952	Splenic sequestration
7891	Hepatomegaly
7892	Splenomegaly
2894	Hypersplenism
572	Liver abscess and sequelae of chronic liver disease
5720	Abscess of liver
421	Acute and subacute endocarditis
4249	Acute and subacute endocarditis
6951	Erythema multiforme (fill)
6952	Erythema nodosum
2870	Allergic purpura

*ICD-9-CM, International Classification of Diseases, 9th revision, clinical modification; NOS, not otherwise specified.
